# An approach to compare genome tiling microarray and MPSS sequencing data for transcript mapping

**DOI:** 10.1186/1756-0500-2-211

**Published:** 2009-10-21

**Authors:** Rajkumar Sasidharan, Ashish Agarwal, Joel Rozowsky, Mark Gerstein

**Affiliations:** 1 Molecular Biophysics and Biochemistry Department, Yale University, New Haven, CT 06520, USA; 2 Department of Plant Biology, Carnegie Institution for Science, Stanford, California 94305, USA; 3 Interdepartmental Program in Computational Biology and Bioinformatics, Yale University, New Haven, CT 06520, USA; 4 Department of Computer Science, Yale University, New Haven, CT 06520, USA

## Abstract

We are correcting the abstract of our published article ([1]). The sentence that starts "We observe that 4.5% of MPSS tags...." was not scientifically complete in the original abstract, having only two of the four numbers required to describe a comparison of two technologies in two different organisms. The abstract below more accurately describes our findings, as documented in Figure 1 of the manuscript.

## Background

There are two main technologies for transcriptome profiling, namely, tiling microarrays and high-throughput sequencing. Recently there has been a tremendous amount of excitement about the latter because of the advent of next-generation sequencing technologies and its promises. Consequently, the question of the moment is how these two technologies compare. Here, we attempt to develop an approach to do a fair comparison of expressed transcripts identified from tiling microarray and MPSS tag sequencing data.

## Findings

This comparison is a challenging task because the sequencing data is discrete while the tiling array data is continuous. We use the published Rice and Arabidopsis datasets which provide currently best matched sets of arrays and sequencing experiments using an earlier generation of sequencing technology, the MPSS tag sequencing approach. After scoring the arrays consistently in both the organisms, a first pass comparison reveals a surprisingly small overlap in expressed transcripts identified using the two technologies. We observe that 4.5% of MPSS tags overlap with 22% of transcripts detected from tiling array data in Rice while 13% of MPSS tags overlap with 66% of transcripts identified from tiling array data in Arabidopsis. However, a closer look at the data suggests that this is an underestimate. When we map tiling array probe intensities onto MPSS sequencing tags and then look at their intensity distribution, we see that the intensity distribution is very similar to exons detected from the respective tiling array data. Furthermore, restricting our comparison to only protein-coding gene loci reveals a very good overlap between the two technologies.

## Conclusion

Our approach to compare genome tiling microarray and MPSS sequencing data suggests that there is actually a reasonable overlap in expressed transcripts identified by the two technologies. This overlap is distorted by the thresholding and scoring strategies employed in the tiling array transcript segmentation procedure.
